# Neural architecture search for pneumonia diagnosis from chest X-rays

**DOI:** 10.1038/s41598-022-15341-0

**Published:** 2022-07-04

**Authors:** Abhibha Gupta, Parth Sheth, Pengtao Xie

**Affiliations:** 1 Department of Computer Science and Engineering, Indian Institute of Information Technology, Nagpur, Nagpur, 441108 India; 2 Department of Electronics and Communication Engineering, Indian Institute of Technology, Guwahati, Guwahati, 781039 India; 3 Department of Electrical and Computer Engineering, University of California, San Diego, San Diego, 92093 USA

**Keywords:** Health care, Medical research, Mathematics and computing

## Abstract

Pneumonia is one of the diseases that causes the most fatalities worldwide, especially in children. Recently, pneumonia-caused deaths have increased dramatically due to the novel Coronavirus global pandemic. Chest X-ray (CXR) images are one of the most readily available and common imaging modality for the detection and identification of pneumonia. However, the detection of pneumonia from chest radiography is a difficult task even for experienced radiologists. Artificial Intelligence (AI) based systems have great potential in assisting in quick and accurate diagnosis of pneumonia from chest X-rays. The aim of this study is to develop a Neural Architecture Search (NAS) method to find the best convolutional architecture capable of detecting pneumonia from chest X-rays. We propose a Learning by Teaching framework inspired by the teaching-driven learning methodology from humans, and conduct experiments on a pneumonia chest X-ray dataset with over 5000 images. Our proposed method yields an area under ROC curve (AUC) of 97.6% for pneumonia detection, which improves upon previous NAS methods by 5.1% (absolute).

## Introduction

Research has shown that deep learning methods are able to obtain human level accuracy in image classification, detection, and segmentation^1^. Motivated by these successes, AI practitioners have explored the effectiveness of these methods in biomedical domains. Deep learning has been used for a wide variety of healthcare applications such as classification and detection of tumors from medical images, making treatment plans by analyzing electronic health records, to name a few. An essential element for the success of deep learning techniques is the capability of neural networks to learn high level abstractions from input raw data through a general purpose learning procedure^2^. Deep learning based clinical systems provide support for experts in the medical domain in performing time-consuming works, such as examining chest radiographs for the signs of pneumonia.

Pneumonia is a life threatening disease caused either by pathogens like bacteria, virus or fungi in the lungs. Pneumonia caused due to viruses is milder as compared to its bacterial counterpart and the symptoms occur gradually. In comparison, bacterial pneumonia is more severe and its symptoms can occur suddenly, especially among groups at high risk, such as children^3^. Bacterial pneumonia affects a large part of the lung by attacking the lobes. A person needs to be hospitalized if the infection spreads to other lobes as well^4^. Fungal pneumonia is a variant which occurs among people having weak immunity. This type of pneumonia can be dangerous as well, and requires time for the patient to regain health. Infants, people having other diseases, people with an impaired immune system, the elderly, people who have a history of hospitalization or are suffering from a chronic disease such as asthma or smokers are some of the groups who are at a high risk of pneumonia. Severe Acute Respiratory Syndrome Coronavirus 2 (SARS-CoV-2) is the pathogen responsible for the Coronavirus disease 2019 (COVID-19) pandemic. The new COVID-19 induced pneumonia causes severe inflammation in lungs. It damages cells and tissues of air sacs in lungs. These sacs are where the oxygen is processed and delivered to the blood. A study conducted by^5^ shows that the mortality rate of patients suffering from COVID-19 induced pneumonia is 56%, showing that severe COVID-19 pneumonia is associated with very high mortality.

There is an urgent need to develop new methods that aid in the effective identification of pneumonia in early stages to reduce patient mortality^6^. In countries which lack medical resources, especially in the rural areas, there is a strong need for computer aided diagnosis systems. These artificial intelligence based systems can help radiologists detect pneumonia from chest X-ray images in early stages.

Several medical tests are used for the detection of pneumonia, such as pulse oximetry, sputum test and chest X-rays. A primary method in the detection of pneumonia is using chest radiographs. In this paper, we propose a Learning by Teaching (LBT) framework to perform differential architecture search to discover the most effective neural architecture for detecting pneumonia from chest X-ray images. We also experiment with other methods for neural architecture search such as DARTS^7^ and PC-DARTS^8^. The models are trained on a dataset consisting of 5215 chest X-ray images, containing 1341 images labeled as ‘Normal’, indicating the CXR images have no abnormalities, and 3874 images as ‘Pneumonia’, indicating bacterial or viral pneumonia. Experiments demonstrate the efficacy of our method which achieves a pneumonia classification AUC of 97.6%. The novelties of our work are twofold. First, to our best knowledge, our work represents the one studying neural architecture search for pneumonia detection from chest X-rays. Second, we propose a three-level optimization framework which uses a student model to improve the search of teacher’s architecture, which is a novel method.

## Methods

In this section, we introduce our proposed LBT method for searching optimal architectures to detect pneumonia. There are no human participants involved in this study.

### Differentiable architecture search (DARTS)

Experiments are carried out using the method proposed by Liu et al.^7^ called DARTS (Differentiable ARchiTecture Search) which is effective in discovering high performance convolutional architectures suitable for image classification. The algorithm searches for a computation cell which is considered as a building block of the final architecture. The searched cell can then be stacked to form a convolutional neural network capable of classifying images. The cell is a Directed Acyclic Graph (DAG) where each directed edge represents an operation such as convolution, pooling, etc. The method performs continuous relaxation of the search space by considering multiple operations on the edges and performing a softmax on them according to Eq. (1),

1 $$\begin{aligned} {\bar{O}}^{(i, j)}(x)=\sum _{o \in {\mathscr {O}}} \frac{\exp \left( \alpha _{o}^{(i, j)}\right) }{\sum _{o^{\prime } \in {\mathscr {O}}} \exp \left( \alpha _{o^{\prime }}^{(i, j)}\right) } o(x), \end{aligned}$$

where ${\mathscr {O}}$ is a set of candidate operations (such as convolution, max pooling, etc.) applied to an intermediate representation ${x}^{(i)}$. $\alpha ^{(i,j)}$ is a vector that depicts the mixing of weights for a pair of nodes (*i*, *j*). The final architecture is induced by performing joint optimization of the network’s weights and architecture. Their method sets itself apart by searching over a continuous search space instead of a discrete search space, so that the architecture can be optimized by minimizing the loss on a validation set using gradient descent. The computational efficiency of gradient-based optimization, as opposed to inefficient black-box search, allows DARTS to achieve competitive performance comparable to the state of the art using orders of magnitude less computation.

### Partial channel connection for memory efficient architecture search (PC-DARTS)

Experiments are also carried out using PC-DARTS (Partially Connected DARTS)^8^. This technique has a considerably lower memory footprint and computational overheads, as compared to DARTS^7^. The core idea behind PC-DARTS is that it randomly selects a subset of channels (determined by a hyperparameter) while bypassing the others. A benefit of this approach is that the search operation becomes more regularized and less prone to reaching a local optima. The algorithm in PC-DARTS applies a masking scheme to sample channels according to Eq. (2).

2 $$\begin{aligned} f_{i, j}^{\mathrm {PC}}\left( {\mathbf {x}}_{i} ; {\mathbf {S}}_{i, j}\right) =\sum _{o \in {\mathscr {O}}} \frac{\exp \left\{ \alpha _{i, j}^{o}\right\} }{\sum _{o^{\prime } \in {\mathscr {O}}} \exp \left\{ \alpha _{i, j}^{o^{\prime }}\right\} } \cdot o\left( {\mathbf {S}}_{i, j} * {\mathbf {x}}_{i}\right) +\left( 1-{\mathbf {S}}_{i, j}\right) * {\mathbf {x}}_{i}, \end{aligned}$$

where $${S}_{i, j}$$ is a channel sampling mask, which uses 1 to select channels and 0 to masked channels. $$ {S}_{i,j}*x_i$$ and $$(1-{S}_{i,j})*x_i$$ denote the selected and masked channels, respectively. The proportion of selected channels is decided by a hyperparameter 1/*K*. The selection of partial channels reduces the memory overhead of computing $$ f_{i, j}^{\mathrm {PC}}\left( {\mathbf {x}}_{i} ; {\mathbf {S}}_{i, j}\right) $$ by *K* times and allows larger batch sizes during the training process. Larger batch sizes ensure stability during the search process. PC-DARTS deal with instability of channel selection across different iterations based on edge normalization. This is achieved by introducing a parameter $${\beta }$$ that adds weights on each edge *(i,j)*. Since $${\beta }_{i,j}$$ is shared through the training process, the learned network architecture is insensitive to the sampled channels across iterations, making the architecture search more stable as compared to DARTS.

### Learning by teaching

Inspired by human learning strategies, we propose a framework called LBT (Learning By Teaching) which improves the learning outcome of a model by encouraging it to teach other models to perform well. The LBT framework is used to perform NAS to determine the best architecture for detecting pneumonia from chest X-ray images.

In our framework, there is a teacher model and a student model. The eventual goal is to make the teacher achieve better learning outcomes. The way to achieve this goal is to let the teacher teach the student to perform well on the target task. The intuition behind LBT is that a teacher needs to learn a topic very well in order to teach this topic to a student clearly. Teaching is performed based on pseudo-labeling^9^: the teacher uses its model to generate a pseudo-labeled dataset; the student is trained on the pseudo-labeled dataset. The teacher has a learnable neural architecture *A* and a set of learnable network weights *T*. The student has a predefined architecture (by humans) and a set of learnable network weights *S*. The teacher has a training dataset $D^{(\text {tr})}_t$ and a validation dataset $D^{(\text {val})}_t$. The student has a training dataset $D^{(\text {tr})}_s$ and a validation dataset $D^{(\text {val})}_s$. There is an unlabeled dataset $D_u$ where pseudo labeling is performed.

In our framework, both the teacher and student perform learning, which is organized into three stages. In the first stage, the teacher fixes its architecture and trains its network weights by minimizing the training loss defined on $D^{(\text {tr})}_t$:

3 $$\begin{aligned} T^*(A) =\text {min}_{T} \; L(T,A,D_t^{(\text {tr})}). \end{aligned}$$

The architecture *A* is needed to calculate the loss on training examples. However, it cannot be updated by minimizing the training loss. Otherwise, a degenerated solution will be produced where *A* has very large capacity to overfit the training examples but will yield poor prediction outcomes on unseen examples. $T^*(A)$ is a function of *A*: a different *A* will result in a different training loss $L(A, T,D_t^{(\text {tr})})$; *T* trained by minimizing $L(A, T,D_t^{(\text {tr})})$ will be different as well.

In the second stage, the teacher teaches a student via pseudo-labeling. Given an unlabeled dataset $D_u=\{x_i\}_{i=1}^N$, the teacher uses its model $T^*(A)$ trained in the first stage to make predictions on $D_u$. Assuming the task is classification with *K* classes, the prediction $f(x_i;T^*(A))$ on $x_i$ would be a *K*-dimensional vector, where the *k*-th element indicates the probability that $x_i$ belongs to the *k*-th class and the sum of elements in $f(x_i;T^*(A))$ is one. Let $D_{pl}(D_u,T^*(A))=\{(x_i,f(x_i;T^*(A)))\}_{i=1}^N$ denote the pseudo-labeled dataset. The network weights *S* of the student is trained on $D_{pl}(D_u,T^*(A))$ and a human-labeled training set $D_{s}^{(\text {tr})}$:

$$\begin{aligned} S^*(T^*(A)) = \text {min}_S \; L(S, D_s^{(\text {tr})} )+\lambda L(S, D_{pl}(D_u,T^*(A))). \end{aligned}$$

where $L(\cdot )$ denotes a cross-entropy loss and $\lambda$ is a tradeoff parameter. $S^*(T^*(A))$ is a function of $T^*(A)$: a different $T^*(A)$ will result in a different pseudo-labeled dataset $D_{pl}(D_u,T^*(A))$ which will render the training loss to be different; a different training loss will result in a different $S^*(T^*(A))$.

In the third stage, the student’s model $S^*(T^*(A))$ trained in the second stage is validated on $D_{s}^{(\text {val})}$. Besides, we also validate the teacher’s model $T^*(A)$ trained in the first stage on $D_{t}^{(\text {val})}$. The validation performances provide feedback on how good the teacher’s architecture *A* is. At this stage, *A* is optimized by minimizing the validation losses:

4 $$\begin{aligned} \text {min}_{A} \; L(T^*(A),A, D_t^{(\text {val})}) + \gamma L(S^*(T^*(A)), D_s^{(\text {val})}), \end{aligned}$$

where $\gamma$ is a tradeoff parameter.

Given the three learning stages, we propose a three-level optimization framework to stitch them together:

5 $$\begin{aligned} \begin{array}{l} \underset{A}{\text {min}} \;\; L(T^*(A),A, D_t^{(\text {val})})) + \gamma L(S^*(T^*(A)), D_s^{(\text {val})})\\ s.t. \;\;\; S^*(T^*(A)) = \underset{S}{\text {min}} \;\; L(S, D_s^{(\text {tr})} )+\lambda L(S, D_{pl}(D_u,T^*(A)))\\ \quad \;\;\;\; T^*(A) = \underset{T}{\text {min}} \; L(A, T,D_t^{(\text {tr})}) \end{array} \end{aligned}$$

The three level optimization problem is solved using a gradient based algorithm.

For computational efficiency, we search *A* in a differentiable way as DARTS^7^: given an overparameterized network, a subnetwork is carved out as the final architecture. The overparameterized network contains a large number of basic building blocks such as convolution operations, pooling operations, etc. The output of each building block is multiplied with a scalar. The search algorithm optimizes these scalars by minimizing validation losses. In the end, building blocks with the largest scalars form the final architecture.

## Dataset

**Figure 1:**
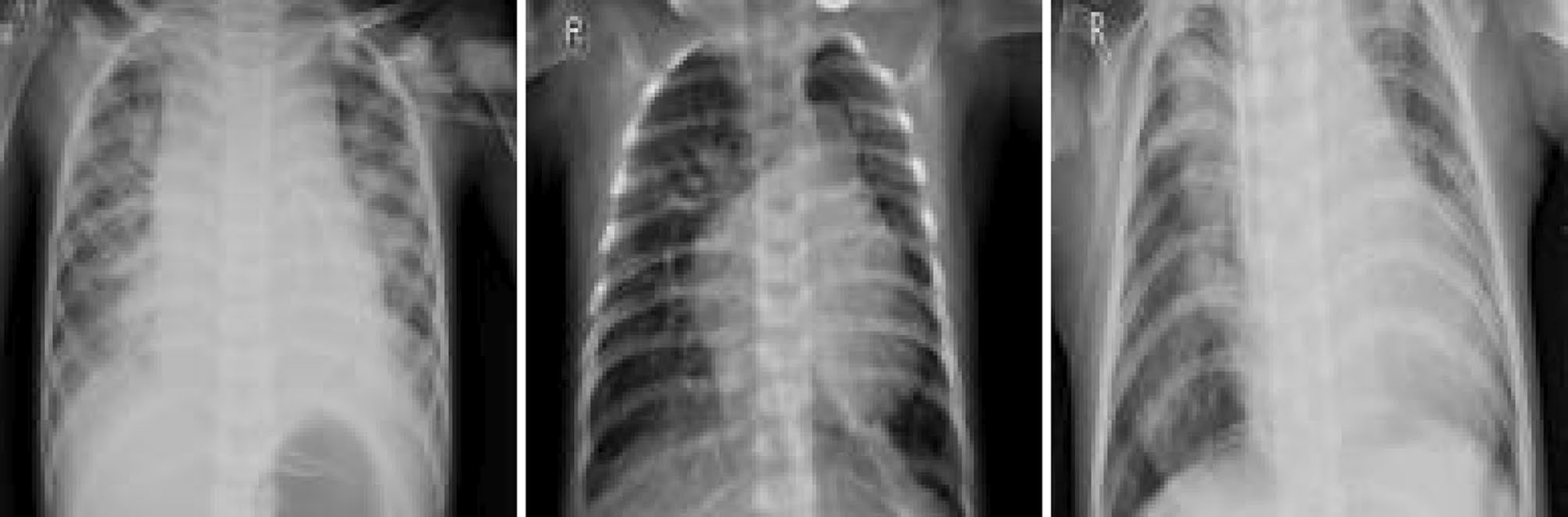
Some randomly sampled X-rays (with a size of 128 × 128) containing pneumonia.

We used the chest X-ray dataset provided by^10^. There are 5,863 chest X-Ray images from two classes: Pneumonia and Normal. The pneumonia X-rays contain both bacterial pneumonia and viral pneumonia. Following^10^, we combine these two types of pneumonia into a single Pneumonia class. The chest X-ray images were procured from pediatric patients aged 1 to 5 years from Guangzhou Women and Children’s Medical Center. The chest X-rays of the patients were performed as part of their routine clinical care. Initial screening of the chest radiographs was performed by removing low quality or unreadable scans. The radiographs were then marked as belonging to a pneumonia infected patient or a normal patient by two expert physicians. To make sure that the process was devoid of annotation errors, a third expert was also involved who checked the annotations. The chest X-rays are resized to $128\times 128$. Figure 1 shows some randomly sampled X-rays containing pneumonia. As can be seen, these images are large enough that the clinical manifestations of pneumonia can be clearly observed. We perform evaluation using fivefold cross validation. We randomly split the dataset into fivefold. We run the following experiments by taking turns on the fivefold: in each run, onefold is used as the test set and the other fourfold are used as the training set. Architecture search and model weights training are performed on the training set (which is split into $D^{(tr)}_t$ and $D^{(val)}_t$ with a ratio of 1:1). The searched architecture and trained model weights are evaluated on the test set. We report the mean and standard deviation of the five test performance numbers.

## Related work

In the past few years, many researchers have proposed different deep learning based methods for lung nodule detection, pneumonia detection and localization, and have curated datasets for these tasks. Rajpurkar et al.^11^ proposed CheXNeXt, a deep CNN consisting of 121 layers and capable of detecting 14 different diseases from chest X-rays, including pneumonia. Their method detects abnormalities in input X-ray images and uses an ensemble of neural networks to calculate mean predictions. Wang et al.^12^ curated a new dataset called ChestX-ray8 containing around 100,000 chest X-rays. They achieved a promising AUC score of 0.63 on detecting pneumonia from CXR images. Wozniak et al.^13^ developed probabilistic neural networks to detect small lung nodules from CXR images. Jung et al.^14^ used a 3D deep CNN with shortcuts and dense connections that tackle the vanishing gradient problem for the detection of lung nodules. Gu et al.^15^ proposed a 3D deep CNN and employed a multi-scale prediction strategy to detect nodules in lungs. They augment test data to detect small nodules. Li et al.^16^ have employed a CNN based approach combined with rib suppression and lung filled segmentation to detect lung nodules using chest radiographs. They trained three networks on images with different resolutions and applied feature fusion to merge information.

Ho et al.^17^ proposed a localization approach using pre-trained DenseNet-121 and a classification based approach that integrates local and deep features to establish state of the art classification results on 14 thoracic diseases on the ChestX-ray14 dataset. Gabruseva et al.^18^ proposed to localize lung opacity regions from X-ray images using RetinaNet^19^ and SE-ResNext101^20^ pre-trained on ImageNet^21^. Souza et al.^22^ investigated the problem of detecting dense abnormalities in chest X-Ray images while performing automatic lung segmentation using two deep CNNs. Their method achieved an accuracy of 96.79%. Xu et al.^23^ tackled the problem of anomaly detection in chest X-rays by designing a new hierarchical CNN structure called CXNet-m1, which is shorter, thinner but more powerful than conventional CNNs. They also developed a loss function which can learn discriminative information from misclassified and indistinguishable images. These methods achieve high F1 scores in anomaly detection. Ronneberger et al.^24^ used data augmentation techniques along with CNN to improve biomedical image segmentation. Jaiswal et al.^25^ used Mask R-CNN^26^ to detect pneumonia from chest radiographs accurately. The model leverages both local and global features and uses dropout and L2 regularization for pneumonia identification. Liang et al.^27^ proposed a deep learning framework that combines residual connection and dilated convolution to diagnose pneumonia. They also proposed methodologies to solve the problem of low image resolution and partial occlusion in CXR images. Sirazitdinov et al.^28^ used an ensemble approach which integrates RetinaNet and Mask R-CNN for pneumonia localization. The network first recognizes regions affected by pneumonia and then non-maximum suppression is applied to the affected regions. Kermany et al.^10^ proposed a transfer learning framework where an Inception V3^29^ architecture was first pre-trained on the ImageNet^21^ dataset and then its softmax layer was trained from scratch to distinguish images containing pneumonia from normal images. Stephen et al.^30^ employ image augmentation techniques to increase the size and quality of pneumonia X-ray data. Siddiqui^31^ proposed a 18-layer deep sequential convolutional neural network consisting of 6 convolutional layers to detect pneumonia from chest X-rays. Gu et al.^32^ used a VGG16^33^ model for pneumonia detection. Their model consists of two parts: a fully convolutional neural network for lung region identification and a deep CNN for classifying pneumonia.

Santosh and Ghosh^34^ performed a systematic analysis of AI-based medical imaging methods for COVID-19 detection from CT and X-rays in terms of dataset size and computational complexity. Santosh and Antani^35^ proposed to leverage lung region symmetry features for automated screening of pulmonary abnormalities from chest X-rays. Santosh et al.^36^ perform edge map analysis of chest X-rays to automatically screen pulmonary abnormality. Das et al.^37^ proposed a truncated inception net for COVID-19 outbreak screening from chest X-rays. Mukherjee et al.^38^ developed a unified deep neural network which leverages CT scans and chest X-rays simultaneously to detect COVID-19.

In a recent method called Meta Pseudo Labels^39^, a teacher model is updated based on the performance of a student model. Our work differs from^39^ in the following aspects. First, our method is based on a three-level optimization framework which searches for teacher’s architecture by minimizing student’s validation loss^39^. is based on two-level optimization which has no architecture search. Second, our method trains the teacher’s network weights before using the teacher to generate pseudo-labels. In contrast^39^, does not train the teacher before using it to perform pseudo-labeling. In the experiments, we compared our method with^39^. Our method outperforms^39^ significantly. Liu et al.^40^ studied unsupervised neural architecture without leveraging human labels. Our work differs from^40^ in two aspects. First, in our method, a teacher network (with a searchable architecture) teaches a student network (with a fixed architecture) via pseudo-labeling. In contrast^40^, has no pseudo-labeling. It searches for an architecture using self-supervised learning, then evaluates this architecture by retraining its weight parameters. Second, our method searches for the teacher’s architecture and trains the student model jointly in an end-to-end framework while^40^ performs architecture search and evaluation separately.

## Experiments

### Data preprocessing

Input images were enhanced before performing architecture search and evaluation. We utilized a simple but effective image enhancement method called Dynamic Histogram Equalization (DHE)^41^ to improve the quality of input images. Benefits of this method include: (1) it does not incur loss of details; (2) it does not introduce severe side effects such as washed-out appearance, checkerboard effects etc., or undesirable artifacts.

### DARTS

Each DARTS experiment consist of two steps, architecture search and architecture evaluation. The first step searches for the optimal cell using DARTS. A cell with the best validation performance is considered as the optimal cell. In the second step, the best cell obtained in the first step is used to construct a larger network, which is trained from scratch and its performance is reported on the test set. The following operations are included in the candidate set *O*: 3 × 3 and 5 × 5 dilated separable convolutions, 3 × 3 and 5 × 5 separable convolutions, 3 × 3 average pooling, 3 × 3 max pooling, identity and zero. If applicable, all the operations involved have stride one. Spatial resolution is preserved by padding convolved feature maps. The ReLU-Conv-BN order is used for convolutional operations, and each separable convolution is always applied twice^42–44^. The convolutional cell has $N=7$ nodes, among which the output node is defined as the depth wise concatenation of all the intermediate nodes (input nodes excluded). The rest of the setup follows^42–44^ where a network is formed by stacking multiple cells together. The first and second nodes of cell *k* are set equal to the outputs of cell $k-2$ and cell $k-1$, respectively, and $1\times 1$ convolutions are inserted as necessary. The reduction cells are the ones that are located at one-third and two-thirds of the total depth of the network, in which all the operations adjacent to the input nodes are of stride two. The architecture encoding therefore is (${\alpha }$-normal, ${\alpha }$-reduce), where ${\alpha }$-normal is shared by all normal cells and ${\alpha }$-reduce is shared by all reduction cells. The search experiments are conducted by running the algorithm for 30 epochs with a batch size of 64. Network weights are optimized using SGD, with an initial learning rate of 0.025 (adjusted using a cosine decay scheduler), a momentum of 0.9, and a weight decay of $3e{-}4$. The loss function is Binary Cross Entropy. The initial number of channels is set to 36 and the network is trained for 600 epochs, with mini-batch size set to 64. These experiments are conducted on A100 GPUs.

### PC-DARTS

Similar to DARTS^7^, the PC-DARTS experiments are also performed in two stages: architecture search and architecture evaluation. The operation space *O* is the same as that in DARTS. An alternative and more efficient implementation is used for partial channel connections. For edge *(i,j)*, channel sampling is not performed at each time of computing *o*(${x_i}$), but instead choosing the first 1/*K* channels of ${x_i}$ for the operation mixture directly. To compensate, after ${x}_{j}$ is obtained, its channels are shuffled before being used for further computations. This is the same as the implementation used in ShuffleNet^45^, which is more GPU-friendly and thus runs faster. The search experiments are conducted by running the algorithm for 30 epochs with a batch size of 128. Network weights are optimized using SGD, with an initial learning rate of 0.025 (adjusted using a cosine decay scheduler), a momentum of 0.9, and a weight decay of $3e-4$. The initial number of channels were set to 36 and the network was trained for 600 epochs, with mini-batch size set to 96.

### LBT

In LBT, for the search space, we experimented with the search spaces defined in DARTS^7^ and PC-DARTS^8^. For the student’s architecture, ResNet-18^46^ is used. ${\lambda }$ and ${\gamma }$ in Eq. (5) are both set to 1. During architecture search, the teacher’s architecture is a stack of 8 cells, each consisting of 7 nodes. The initial channel number is set to 16. The algorithm runs for 50 epochs with a batch size of 32 for LBT-DARTS and 64 for LBT-PC-DARTS. Network weights are optimized using SGD, with an initial learning rate of 0.025 (adjusted using a cosine decay scheduler), a momentum of 0.9, and a weight decay of $3e-4$. The experiments are conducted with different values of $\lambda$. At $\lambda$ = 1, the highest accuracy is obtained. During architecture evaluation, 20 copies of the optimal cell searched in the search phrase are stacked into a large network, which is trained using the combined training and validation datasets. The loss function is Binary Cross Entropy. The initial channel number is set to 40. The network is trained for 600 epochs, with mini-batch size set to 32 for LBT-DARTS and 96 for LBT-PC-DARTS. The experiments are conducted on a Nvidia GeForce GTX 1080Ti GPU.

## Results and discussion

We use sensitivity, specificity, F1, area under ROC curve (AUC), accuracy to measure performance. The results are shown in Table 1. From these two tables, we make the following observations. *First*, among all methods in these two tables, our proposed LBT-PC-DARTS achieves the best performance on all evaluation metrics, with an AUC score of 97.6% and an F1 score of 97.1%. This shows that our method is highly effective in accurately detecting pneumonia from chest X-rays. We performed a two-sided paired Students’ t test between our method and each baseline. We used this test method because the following assumptions are satisfied: (1) the means of two populations (one for our method and the other for a baseline) of performance numbers being compared follow normal distribution; (2) the sample sizes in the two populations are equal (which is the number of fold in cross validation); (3) the data used to perform the test is fully paired: the two populations of performance numbers are evaluated on the same test set in each fold of the fivefold cross validation; (4) two-sided test is used because our method may perform either better or worse than a baseline. In these tests, the p-values are smaller than 0.001, which demonstrates that the improvements of our method over baselines are statistically significant. The reason that our method works better than baselines is as follows. In our method, the teacher model improves its learning ability by teaching a student model to perform well on the classification task. The student is trained on the pseudo-labeled dataset created by the teacher. If the student does not perform well on the validation set, that means the pseudo labels are not correct, which indicates the teacher’s model is not accurate. To avoid such an outcome, the teacher enforces itself to learn better to generate correct pseudo labels. *Second*, while our LBT-PC-DARTS method achieves better performance than baselines, it has a smaller model size than baselines. A smaller model consumes less memory and facilitates faster computation. *Third*, when our LBT is applied to DARTS and PC-DARTS, both of them are improved. This shows that our method is broadly effective to improve different NAS methods. *Fourth*, LBT-PC-DARTS is more effective than LBT-DARTS. For example, the AUC of LBT-PC-DARTS is 2.7% (absolute) higher than LBT-DARTS. LBT-PC-DARTS randomly samples a proportion of channels for operation search. Consequently, it is more memory efficient and allows a larger batch size to be used for higher stability, as compared to LBT-DARTS. In LBT-PC-DARTS, an additional contribution to search stability is made by edge normalization, a light-weighted module that requires no extra computation. *Fifth*, our LBT-PC-DARTS method performs better than transfer learning methods which use pre-trained models, such as InceptionV3^29^, Densenet 121^47^, VGG16^33^, VGG19^33^, Xception^48^, GoogLeNet^49^ and AlexNet^50^, with significantly smaller model size. All these models were pre-trained on large datasets such as ImageNet^21^ and fine-tuning was carried out by freezing the initial layers and training the classification layers from scratch. *Sixth*, our LBT-PC-DARTS method outperforms several state of the art methods^10,27,30,31^ developed for pneumonia detection, with smaller model size. We further conclude that the architecture searched by our framework is lighter and more effective for pneumonia detection. *Seventh*, our LBT-PC-DARTS method has smaller training cost and inference time than baselines while our method achieves better classification performance.

We also performed a human evaluation where our methods are compared with three junior radiologists. From a teaching hospital in Beijing, China, we obtained 50 chest X-rays that have pneumonia and 50 chest X-rays which do not have pneumonia. These X-rays are randomly selected from the hospital’s database and their labels (whether having pneumonia or not) are given by senior radiologists who have more than 20 years of experience of interpreting chest X-rays. We compared our method with three licensed radiologists who have at least 5 years of experience of interpreting chest X-rays. For each of the 100 X-rays (which were randomly shuffled), each junior radiologist judged whether it contains pneumonia. Different radiologists made judgments independently. Table 2 shows the accuracy (since the number of examples in the pneumonia class and normal class are balanced, we did not measure metrics for imbalanced classification, including sensitivity, specificity, F1, and AUC). As can be seen, the performance of our LBT-PC-DARTS method is on par with the three junior radiologists. Besides, our LBT-PC-DARTS method achieves better accuracy than the baselines.

**Table 1:** Comparison between our method and baselines.

Model	Sensitivity (%)	Specificity (%)	F1 (%)	AUC (%)	Accuracy (%)	Model size	Training time (h)	Inference time (ms)
VGG19^51^	92.7 ± 0.68	92.4 ± 0.93	93.0 ± 0.59	93.9 ± 0.81	92.7 ± 0.84	731	2.3	69.2
InceptionV3^52^	91.8 ± 0.49	92.2 ± 0.70	91.4 ± 0.76	92.8 ± 0.55	92.6 ± 0.92	502	2.1	38.6
DenseNet121^52^	93.8 ± 0.87	91.7 ± 0.92	92.4 ± 0.96	93.8 ± 0.53	93.1 ± 0.97	537	2.1	87.2
AlexNet^52^	92.5 ± 1.04	92.7 ± 0.85	92.1 ± 0.62	94.1 ± 0.62	92.7 ± 0.73	433	2.0	32.7
VGG16^51^	90.9 ± 0.75	94.1 ± 0.68	91.8 ± 1.15	94.3 ± 0.47	92.5 ± 0.61	737	2.2	55.3
Xception^51^	90.7 ± 0.59	92.3 ± 0.71	93.6 ± 0.74	93.4 ± 0.62	92.1 ± 0.73	241	1.8	146.9
GoogLeNet^52^	90.7 ± 1.03	92.5 ± 0.91	91.8 ± 0.72	95.4 ± 0.37	93.4 ± 0.85	87	1.5	38.1
LeNet5^53^	84.6 ± 0.72	85.9 ± 0.55	85.4 ± 0.59	88.7 ± 0.36	89.1 ± 0.42	11.1	0.2	28.0
Kermany et al.^10^	92.8 ± 0.59	92.2 ± 0.57	92.5 ± 0.96	93.7 ± 0.69	93.0 ± 0.68	403	2.2	172.6
Stephen et al.^30^	92.4 ± 0.71	92.7 ± 0.38	92.4 ± 0.96	94.2 ± 0.71	93.7 ± 0.62	61	1.6	147.0
Siddiqi^31^	94.7 ± 0.42	93.1 ± 1.33	92.7 ± 0.61	93.9 ± 0.33	93.5 ± 0.74	274	1.7	210.6
Liang et al.^27^	89.5 ± 0.62	91.7 ± 0.73	89.9 ± 1.04	92.2 ± 0.68	92.3 ± 0.95	$\approx$ 215	1.9	187.3
Meta Pseudo Label^39^	90.6 ± 0.74	92.3 ± 0.58	91.7 ± 0.94	93.2 ± 0.63	91.8 ± 0.71	69	1.7	162.5
Liu et al.^40^	92.0 ± 0.58	92.7 ± 0.84	92.4 ± 0.81	93.4 ± 0.45	92.4 ± 0.74	35	1.4	85.2
Kundu et al.^54^	92.4 ± 1.05	91.6 ± 0.69	91.8 ± 0.95	93.1 ± 0.52	91.9 ± 0.50	195	2.1	141.7
Cha et al.^55^	92.1 ± 0.62	91.3 ± 0.62	91.4 ± 0.77	93.2 ± 0.68	92.0 ± 0.59	131	1.7	196.3
DARTS^7^	88.9 ± 0.71	89.2 ± 0.95	90.1 ± 0.62	93.0 ± 0.85	89.8 ± 0.75	11.4	0.9	28.7
LBT-DARTS (ours)	93.0 ± 0.42	93.2 ± 0.86	92.8 ± 0.75	94.9 ± 0.82	93.3 ± 0.61	11.2	0.9	28.5
PC-DARTS^8^	93.2 ± 0.84	90.9 ± 0.95	91.8 ± 0.62	92.5 ± 0.60	91.4 ± 0.75	11.3	0.1	28.5
LBT-PC-DARTS (ours)	**95.9** ± 0.74	**96.7** ± 0.92	**97.1** ± 0.64	**97.6** ± 0.58	**97.0** ± 0.80	**10.9**	0.1	**26.4**

Model size is in MB. Training time is in GPU hours (h). Inference time is in milliseconds (ms).

Significant values are in bold.

**Table 2:** Comparison between our method and three junior radiologists.

	Accuracy (%)
VGG19^51^	89.7
InceptionV3^52^	90.4
DenseNet121^52^	91.1
AlexNet^52^	89.2
VGG16^51^	90.5
Xception^51^	89.6
GoogLeNet^52^	88.4
LeNet5^53^	82.0
Kermany et al.^10^	91.0
Stephen et al.^30^	90.9
Siddiqi^31^	91.3
Liang et al.^27^	88.7
Meta Pseudo Label^39^	89.1
Liu et al.^40^	90.4
Kundu et al.^54^	91.2
Cha et al.^55^	90.7
DARTS^7^	88.5
LBT-DARTS (ours)	91.8
PC-DARTS^8^	89.2
LBT-PC-DARTS (ours)	94.2
Radiologist 1	94.5
Radiologist 2	94.7
Radiologist 3	94.4

## Ablation studies

In this section, we perform ablation studies to better understand the individual ingredients in our proposed method.

### Ablation setting 1

In this setting, the teacher updates its architecture by minimizing the validation loss of the student only, without considering the validation loss of itself. The corresponding formulation is outlined in Eq. (6). In this study, $\lambda$ is set to 1. The student’s architecture is ResNet-18.

6 $$\begin{aligned} \begin{array}{ll} \min _{A} &{} L\left( S^{*}\left( T^{*}(A)\right) , D_{s}^{(\mathrm {val})}\right) \\ \text{ s.t. } &{} S^{*}\left( T^{*}(A)\right) =\min _{S} L\left( S, D_{s}^{(\mathrm {tr})}\right) +\lambda L\left( S, D_{p l}\left( D_{u}, T^{*}(A)\right) \right) \\ &{} T^{*}(A)=\min _{T} L\left( A, T, D_{t}^{(\mathrm {tr})}\right) \end{array} \end{aligned}$$

### Ablation setting 2

In this setting, in the second stage of LBT, only the pseudo labeled dataset is used to train the student. The training data of the student, labeled by humans, is not used. The corresponding formulation is outlined in Eq. (7). In this study, $\gamma$ is set to 1. The student’s architecture is ResNet-18.

7 $$\begin{aligned} \begin{array}{ll} \min _{A} &{} L\left( T^{*}(A), A, D_{t}^{(\mathrm {val})}\right) +\gamma L\left( S^{*}\left( T^{*}(A)\right) , D_{s}^{(\mathrm {val})}\right) \\ \text{ s.t. } &{} S^{*}\left( T^{*}(A)\right) =\min _{S} L\left( S, D_{p l}\left( D_{u}, T^{*}(A)\right) \right) \\ &{} T^{*}(A)=\min _{T} L\left( A, T, D_{t}^{(\mathrm {tr})}\right) \end{array} \end{aligned}$$

### Ablation setting on $\lambda$

We investigate how the teacher’s test error changes with the tradeoff parameter $\lambda$. In this study, the other tradeoff parameter $\gamma$ is set to 1. Architecture search is performed on the training and validation sets. Architecture evaluation results are reported on the test set. The student’s architecture is ResNet-18.

### Ablation setting on $\gamma$

We investigate how the teacher’s test error changes with the tradeoff parameter $\gamma$. The other tradeoff parameter $\lambda$ is set to 1. Similar to the ablation study on $\lambda$, the error is reported on the test set. The student’s architecture is ResNet-18.

**Table 3:** Ablation studies.

Ablation studies	Accuracy (%)
LBT-PC-DARTS (ours)	**97.0**
Ablation setting 1	94.3
Ablation setting 2	95.1

Significant values are in bold.

### Results

**Figure 2:**
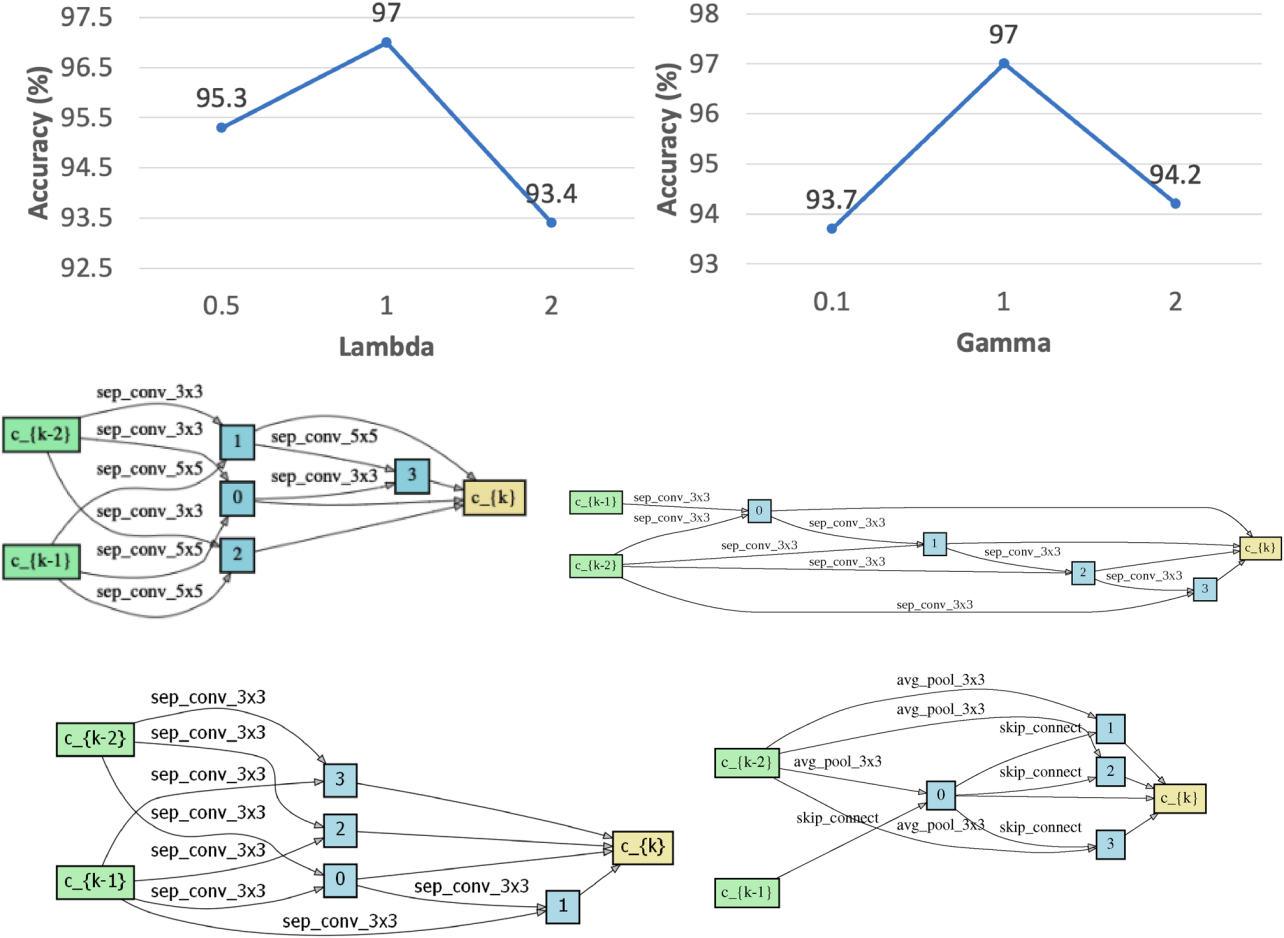
Top row: accuracy of LBT-PC-DARTS under different values of the tradeoff parameter ${\lambda }$ and ${\gamma }$. Middle row: normal cell (left) and reduction cell (right) searched by LBT-DARTS. Bottom row: normal cell (left) and reduction cell (right) searched by LBT-PC-DARTS.

Table 3 shows the performance of LBT-PC-DARTS for ablation setting 1 and 2. Figure 2 shows how the accuracy of LBT-PC-DARTS changes with the tradeoff parameters $\lambda$ and $\gamma$.

In ablation setting 1, only the student’s validation loss is leveraged to update the architecture. It can be observed that there is a 2.7% (absolute) drop in accuracy as compared to the full LBT-PC-DARTS setting where both the student’s validation loss and the teacher’s validation loss are leveraged. The reason is that a student’s validation loss indirectly measures the quality of the teacher’s architecture. How well the student performs depends on not only how well the teacher teaches the student but also how strong the student itself is. If the student is a very strong learner, its validation loss may be largely determined by the student itself and less influenced by the teacher. In this case, student’s validation would be a relatively weak signal for guiding the learning of the teacher. In contrast, the validation loss of the teacher directly depends on its architecture and can serve as a direct (hence strong) signal to guide the teacher to learn. In the end, combining the direct signal (teacher’s validation loss) and indirect signal (student’s validation loss) together is more beneficial than using the indirect signal only.

Ablation setting 2 incurs a 1.9% decrease in accuracy compared with our full LBT-PC-DARTS method. In other words, using both the pseudo-labeled dataset and human-labeled dataset to train the student yields better performance than using the pseudo-labeled dataset only. The reason is that since the pseudo-labels are automatically generated by a model, they are not entirely reliable. Trained on less reliable labels, the student’s model may have low quality and a poorly-performing student cannot drive the teacher to learn better. This risk can be reduced by incorporating human-provided labels which are more reliable. As a result, using human labels and pseudo-labels jointly yields better performance than solely using pseudo-labels.

In Fig. 2 (top row, left), how the classification accuracy of LBT-PC-DARTS changes with $\lambda$ is shown. We can make several observations from this figure. When we increase the value of $\lambda$ from 0.5 to 1, there is a 1.7% (absolute) improvement in accuracy. This is because a larger $\lambda$ incurs a stronger effect of teaching, where the training of the student relies more on the pseudo-labeled dataset created by the teacher. When the teaching effect is strong, the teacher can gain more feedback from the student’s performance, which helps the teacher to learn better. On the other hand, further increasing the value of $\lambda$ leads to a 3.6% (absolute) decrease in performance. The reason is that if $\lambda$ is too large, the teaching effect would be excessively strong. Under such circumstances, the student is mainly trained on the pseudo labels which are less reliable than human-provided labels and consequently its model may be of low quality. A mediocre student will not be very helpful in driving the teacher to improve.

In Fig. 2 (top row, right), how the classification accuracy of LBT-PC-DARTS changes with $\gamma$ is shown. As we increase the value of $\gamma$ from 0.1 to 1, there is a 3.3% (absolute) improvement in accuracy. This is because a larger $\gamma$ encourages the teacher to pay more attention to the feedback obtained from the student. This feedback is valuable because the validation performance of the student reflects the correctness of the pseudo-labels generated by the teacher and the quality of pseudo-labels reflects the quality of the teacher’s architecture. Paying more attention to such feedback enables the teacher to identify its weakness and strive for improvement. On the other hand, further increasing the value of $\gamma$ leads to a 2.8% (absolute) decrease in accuracy. The reason is that if $\gamma$ is too large, the learning of the teacher’s architecture would be guided excessively by the student’s validation loss which is an indirect (hence weaker signal) but inadequately influenced the validation loss of the teacher itself which is a direct (hence stronger signal).

## Visualization

Figure 2 (middle row) and (bottom row) show the cells searched by LBT-DARTS and LBT-PC-DARTS, including normal cells and reduction cells, which form the final architecture in the following way. 20 cells (including normal and reduction cells) are stacked to form the final network. Reduction cells are located at the 1/3 and 2/3 of the total depth of the final network. The rest of the cells in the network are normal cells.

**Figure 3:**
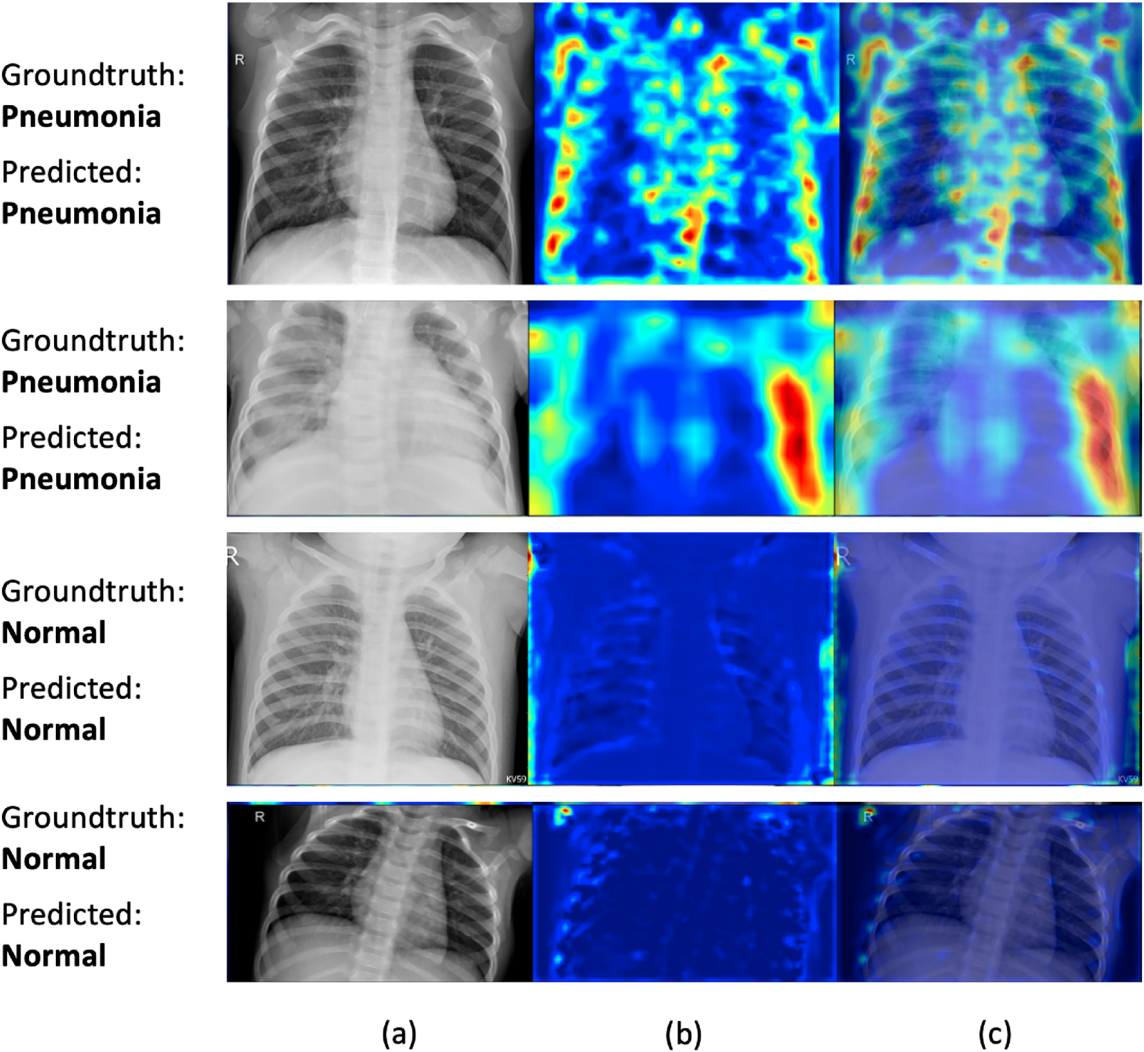
Column (**a**) shows original CXR images. Column (**b**) shows Grad-CAM visualization of saliency maps of LBT-PCDARTS. Column (**c**) shows the overlay of saliency maps on original images.

**Figure 4:**
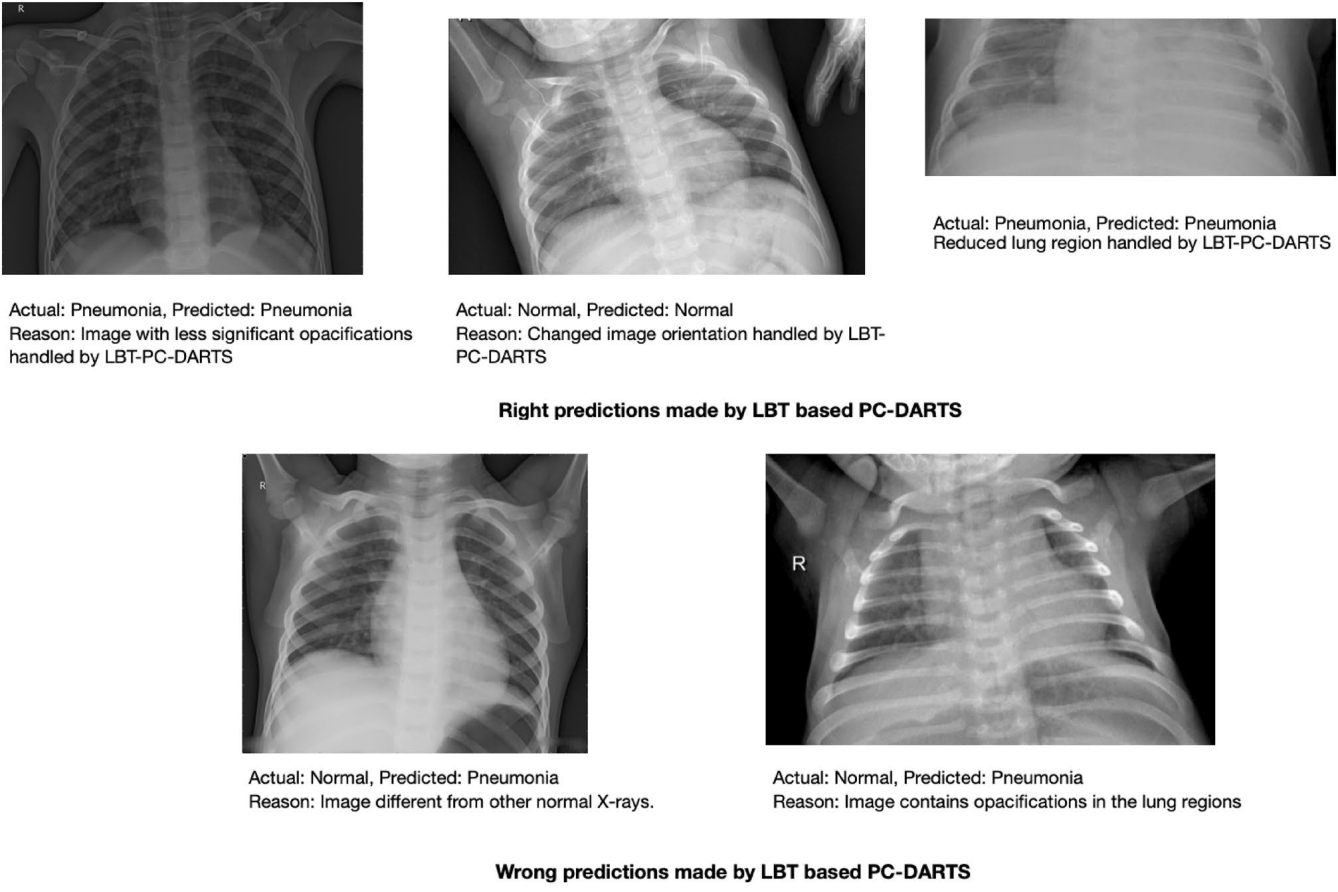
Correct and incorrect predictions made by LBT based PC-DARTS.

**Figure 5:**
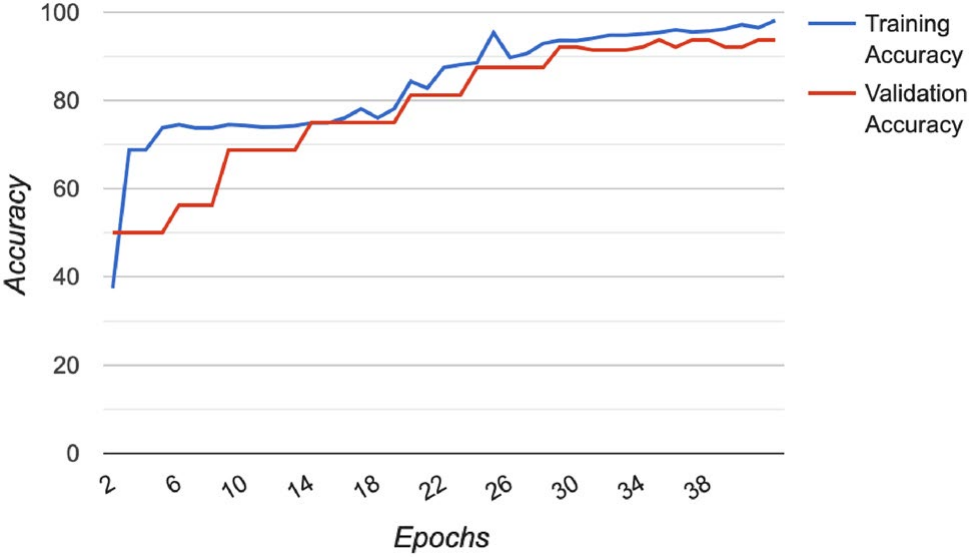
Train and validation accuracy values across epochs during the training process of LBT based PC-DARTS.

Figure 3 shows Grad-CAM^56^ visualization of saliency regions of our methods. As can be seen, for X-rays containing pneumonia, our method identifies correct pneumonia-related regions (highlighted using warm colors) instead of artifacts such as medical device related regions. For normal X-rays, the Grad-CAM visualizations of our method contain little warm colors, which indicates that our method “thinks” these images contain no saliency regions related to pneumonia, which is sensible. Figure 4 shows some correct and incorrect predictions made by LBT based PC-DARTS on the test set. Figure 5 shows the training and validation accuracies across epochs for LBT-PC-DARTS. It can be observed that both training accuracy and validation accuracy steadily improve.

## Conclusion

In this article, the aim is to propose an effective NAS based approach to detect pneumonia from chest radiographs. Experiments are carried out with DARTS, PC-DARTS and LBT based DARTS/PC-DARTS. LBT based PC-DARTS performs the best with an AUC of 97.6%. The proposed framework’s performance is tested against various ablation settings. The results suggest that LBT based NAS methods have great potential in assisting physicians for making accurate diagnosis of pneumonia.

## Data Availability

All experiments are carried out using the publicly available chest X-ray images (with pneumonia) dataset on Kaggle^10^.
